# Temporomandibular Disorders Slow Down the Regeneration Process of Masticatory Muscles: Transcriptomic Analysis

**DOI:** 10.3390/medicina57040354

**Published:** 2021-04-07

**Authors:** Cinzia Sindona, Michele Runci Anastasi, Luigi Chiricosta, Agnese Gugliandolo, Serena Silvestro, Placido Bramanti, Piero Cascone, Emanuela Mazzon

**Affiliations:** 1 IRCCS Centro Neurolesi “Bonino-Pulejo”, 98124 Messina, Italy; cinzia.sindona@irccsme.it (C.S.); michele.runci@irccsme.it (M.R.A.); luigi.chiricosta@irccsme.it (L.C.); agnese.gugliandolo@irccsme.it (A.G.); serena.silvestro@irccsme.it (S.S.); placido.bramanti@irccsme.it (P.B.); 2 Unit of Maxillofacial Surgery, Policlinico Umberto I, “Sapienza” University of Rome, 00161 Rome, Italy; piero.cascone@uniroma1.it

**Keywords:** temporomandibular disorder, migraine, masticatory muscles, RNA-seq analysis, next generation sequencing

## Abstract

*Background and Objectives:* Musculoskeletal injuries represent a pathological condition due to limited joint motility and morphological and functional alterations of the muscles. Temporomandibular disorders (TMDs) are pathological conditions due to alterations in the musculoskeletal system. TMDs mainly cause temporomandibular joint and masticatory muscle dysfunctions following trauma, along with various pathologies and inflammatory processes. TMD affects approximately 15% of the population and causes malocclusion problems and common symptoms such as myofascial pain and migraine. The aim of this work was to provide a transcriptomic profile of masticatory muscles obtained from TMD migraine patients compared to control. *Materials and Methods*: We used Next Generation Sequencing (NGS) technology to evaluate transcriptomes in masseter and temporalis muscle samples. *Results*: The transcriptomic analysis showed a prevalent downregulation of the genes involved in the myogenesis process. *Conclusions*: In conclusion, our findings suggest that the muscle regeneration process in TMD migraine patients may be slowed, therefore therapeutic interventions are needed to restore temporomandibular joint function and promote healing processes.

## 1. Introduction

Temporomandibular disorders (TMDs) represent a group of conditions responsible for pain and dysfunction of the masticatory muscles and temporomandibular joints (TMJs) [1,2]. TMDs affect 10–15% of the adult population aged 20–40 and in prevalence affects women more frequently compared to men [3]. Limited jaw movements, regional pain and acoustic sounds from TMJs during motion are the most frequent hallmarks of TMD [4]. Moreover, morphological and functional alterations of the masticatory muscles, mandibular disarticulation, orofacial pain were reported [2]. A repetitive jaw muscle activity characterized by clenching or grinding of the teeth, and/or bracing or thrusting of the mandible induce a condition known as bruxism [5]. Recently, it was demonstrated that bruxism is considered one of the risk factors that could contribute to progression of TMD [6]. Indeed, this risk factor could compromise the correct functionality of the stomatognathic system and trigger orofacial pain [5]. Additionally, stress, depression, catastrophizing and anxiety are psychosocial conditions that can negatively affect the onset of pain, and thus prolong TMD. Therefore, the TMD may also impact physical and psychosocial functioning, consequently influencing the quality of life of those suffering from these disorders [7].

The cause of TMD may be due to TMJ dysfunctions, and in particular to the antero-medially displacement of the articular disc, that in turn causes friction between the mandibular condyle and the temporal bone with the interposition of retro-discal tissue, which is not used for this function. Therefore, it can tear and cause pain sensation [8,9]. Usually, the drug therapy chosen to treat TMD involves the administration of non-specific drugs such as non-steroidal anti-inflammatory drugs (NSAIDs), various analgesics, and antidepressants; benzodiazepines are also administered for a short time. Otherwise, in the most severe cases, surgical treatments can be found useful to restore TMJ dysfunction and relieve symptoms [3]. Specifically, arthroplasty is a surgical technique that consists of the removal of the superficial part of the mandibular condyle by piezo-surgery (condylar shaving) and repositioning of the articular disc. The goal is discopexy of the articular disc which is done with the lateral ligament by means of a resorbable anchor screw. This technique reduces the friction between the bones and the structure of the TMJ return to normal, useful for relieving the pain sensation and restoring the correct function of masticatory muscles [10].

Several studies highlighted the link between migraine pain and TMD [11,12]. Indeed, migraine and TMD are comorbid disorders, and the presence of one of these conditions increases the prevalence of the other. A possible explanation for this association is the involvement of the same nociceptive system, related to the trigeminus [13]. Trigeminal nerve sensitization triggers migraine pain followed by central or extra-trigeminal sensitization in areas distant from the pain site. Therefore, given the comorbidity between TMD and migraine, the combined therapy showed promising and effective results compared to the single treatment of only one of these conditions [14].

Interestingly, TMJ dysfunction mainly impairs the functionality of the masseter and temporalis muscles [8,9]. Indeed, a painful stimulus is triggered after muscle palpation of the masticatory muscles and, specifically, the masseter shows a greater sensitivity to pain than other masticatory muscles [15]. Furthermore, in masticatory muscles, an association between pain and muscle hardness was observed due to excessive muscle contraction [16]. Of note, Kang et al. have already focused their attention on myofascial pain in TMD patients suffering from migraines, showing that pain is mostly localized in the masseter and temporalis muscle [17].

For these reasons, our work focused on the analysis of temporalis and masseter muscles obtained from muscle biopsies of TMD patients suffering from migraine. The aim of our study was to evaluate the transcriptomic profile of masticatory muscle obtained from TMD patients with migraine in order to assess the molecular mechanism involved in muscles. The transcriptome was obtained using Next Generation Sequencing (NGS), to observe the different mRNAs expression in TMD patients suffering from migraines compared to control. Diagnostic support for our investigation was the compilation of visual analogue scale (VAS) 0–10 questionnaires, useful for evaluating various aspects such as the intensity of perceived pain, symptoms, drug therapy, lifestyle, and other pathologies [10,18,19].

## 2. Materials and Methods

### 2.1. Patients Enrollment

TMD patients with migraine were enrolled for maxillofacial surgery. The protocol was approved by the local Ethics Committee (prot. n. 33/2020 approved on 30 July 2020). TMD diagnosis was confirmed with 3.0 T Magnetic Resonance Imaging (MRI). VAS score was used to classify the degree of migraine. The inclusion criteria were the following: patients aged between 18 and 60 years; confirmed clinical diagnosis; written informed consent obtained from the patient; patients who have no contraindications to perform MRI. The exclusion criteria were the following: proven diagnosis of multiple pathologies; psychiatric diseases with psychotic symptoms; alcohol and drug abuse.

### 2.2. Tissue Collection

Masseter and temporalis muscle biopsies were obtained from four TMD patients suffering from migraine, and one patient with pleomorphic adenoma of the parotid represented the control. The patients underwent general nasotracheal anesthesia. Pre-tragic or retro-tragic preauricular incision is performed, extended by 1–2 cm in the temporal region. The dissection will start to expose the deep temporal fascia and the exposition of the zygomatic arch. Once the deep fascia of the temporalis muscle is reached, a dissection of the tissue downwards by exposing the zygomatic arch is performed. This is a safety plan, while the frontal branch of the facial nerve runs on the superficial fascia, in correspondence with the zygomatic arch. During this surgery, small samples of temporalis muscle and masseter were taken with a minimally invasive procedure. The goal is to detach the parotid gland from the perichondrium that covers the cartilage of tragus and the external auditory canal in all its depth, and to detach the parotid from the temporomandibular joint capsule. The superficial temporal artery and vein are identified, carefully tied and interrupted. Once the vascular structures have been interrupted we can carry on the dissection. Now, by the blunt method, the surgeon goes in search of the joint capsule previously identified as palpatory in order to expose it. The lateral ligament’s insertion is identified on the lateral pole of the condylar head. With a cold blade the insertion is made, the disk is softly moved upward and the inferior compartment of the temporomandibular joint is exposed. The superior compartment of the temporomandibular joint must never be incised in this surgical procedure. To minimize the exposition and to carefully preserve all the functional temporomandibular joint structures, we perform a condylar shaving with piezo-surgery. The goal is to reposition the articular disc. An invitation hole is then performed on the postero-lateral face of the condyle. The discopexy of the articular disc is now performed with the lateral ligament by means of a resorbable anchor screw. In this way, a large part of the contiguity of the joint capsule will be reconstituted [10].

### 2.3. Total RNA Extraction and cDNA Library Preparation

RNA was obtained using the Maxwell^®^ RSC simplyRNA tissue Kit (Promega, Madison, WI, USA) following the manufacturer’s instructions. The library preparation was carried out according to the TruSeq RNA Exome protocol (Illumina, San Diego, CA, USA) following the instructions, as previously reported by Silvestro et al. [20].

### 2.4. Differential Expression Analysis

The data in “Fastq” format were retrieved by the sequencer and the quality of the reads was confirmed using fastQC. The low quality bases and adapters were trimmed with Trimmomatic (Usadel Lab, Aachen, Germany) [21] (version 0.38) and then the reads were aligned and sorted to the human reference genome GRCh38 using Spliced Transcripts Alignment to a Reference (STAR) RNA-seq aligner [22]. The differential expressed analysis was made using htseq-count [23] under python (version 2.7.15, Python Software Foundation, Wilmington, DE, USA) and DESeq2 [24] in R. All the genes with q-value lower than 0.05 after Benjamini-Hochberg correction and absolute fold change lower than 2 were discarded.

## 3. Results

### 3.1. Anamnestic Data

Anamnestic data were collected by completing a questionnaire submitted to patients summarized in Table 1. Enrolled TMD patients 1 and 2 suffered from moderate migraine. TMD patients 3 and 4 suffered from severe migraine, while patient 5 presented pleomorphic adenoma of the parotid and represents control. The mean age of the participants was 61.8 ± 6.72 years (60% male; 40% female).

### 3.2. Groups Comparison Analysis

**Figure 1:**
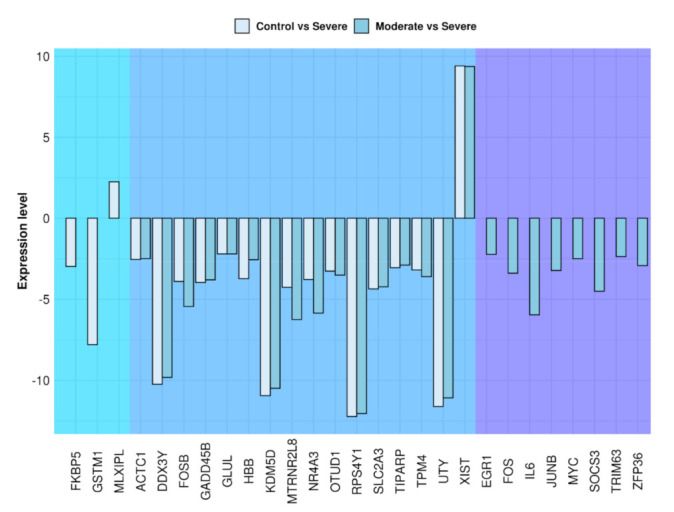
Differently expressed genes in the control or moderate groups against severe. The bar-plot shows that most of the genes are commonly deregulated between the two analysis (center blue frame) with similarly level of expression comparing control (grey bars) or moderate (azure bars) against severe groups. Moreover, eight genes are downregulated only in the moderate against severe analysis (right violet frame) and only three genes are deregulated comparing control against severe group (left azure frame).

In the analysis, we compared the control against the severe groups and the moderate against the severe groups. In Table 2, the 27 genes that statistically differ in the analysis are shown. Among these, 16 genes share the same deregulation between the two analyses. In detail, 15 of them (*ACTC1*, *DDX3Y*, *FOSB*, *GADD45B*, *GLUL*, *HBB*, *KDM5D*, *MTRNR2L8*, *NR4A3*, *OTUD1, RPS4Y1*, *SLC2A3*, *TIPARP*, *TPM4*, *UTY*) are downregulated and only one (*XIST*) is upregulated. Moreover, two genes (*FKBP5*, *GSTM1*) are downregulated and one gene (*MLXIPL*) is upregulated only in the comparison of severe against control. On the other hand, eight genes (*EGR1*, *FOS*, *IL6*, *JUNB*, *MYC*, *SOCS3*, *TRIM63*, *ZPF36*) are downregulated only in the analysis in which the moderate and the severe groups are involved. Figure 1 shows that the moderate group has a greater number of different genes than the control. In addition, most of the genes are downregulated and in common between the two analyses and they are similarly expressed, except for *HBB* that is more downregulated against the control and *FOSB*, *MTRNR2L8*, and *NR4A3* that are more downregulated in the moderate group.

## 4. Discussion

TMD is the generic term used for many clinical features of the masticatory system, such as the masticatory muscles and the TMJs [25]. Often these disorders are related to migraine conditions [26]. In our sample, TMD subjects suffering from migraines were compared to control. All subjects were given the VAS questionnaire (Table 1), useful in assessing migraine intensity based on the frequency of migraine attacks and other symptoms. The analysis of the data allowed the classification of patients with moderate and severe migraines. The aim of the present study was to observe the transcriptomic profile of the masticatory muscles, in particular of the masseter and temporalis muscles, in TMD patients suffering from moderate or severe migraines. Transcriptomic analysis revealed 27 genes that showed a significant fold change, and in particular 23 genes were downregulated and four genes were upregulated. In the immediate comparison of the fold changes, we found, in TMD patients suffering from severe migraine, a significant difference in the genes involved in the myogenesis process. Under physiological conditions, the undifferentiated cells known as satellite cells (SCs) are in a quiescent state in adult skeletal muscle [27] and begin their proliferation after muscle injury. The SCs are located among the plasma membrane of the muscle fiber and the basal lamina. The SCs migrate and proliferate after muscle injury. In myogenesis, there is a proliferation of undifferentiated cells, called myoblasts, which differentiate into myocytes, and consequently merge with other multicellular fibers, reconstituting the mature muscle fiber [28].

Therefore, of considerable interest in our transcriptome is the downregulation of immediate-early genes (IEG) including *FOS*, *FOSB*, and *EGR1* involved in the smooth muscle cells’ (SMCs) proliferation and migration [29].

In detail, in vivo experiment by Miano et al. induced balloon vascular injury in the rat, and analysis of mRNA expression revealed Fos in SMC nuclei, which modulates smooth muscle cell proliferation [30]. Instead, Ramachandran et al. revealed that FosB in the AP-1 subunit is activated as a result of mechanical impulses [31]. Although *EGR1* encodes the transcription factor, it represents another mechano-sensor that induces the formation and repair of tendons during the healing process [32]. Thus, it was possible to deduce that downregulation of these genes in TMD and migraine conditions could negatively affect the proliferative process. *MYC*, usually involved in the myoblasts proliferation and differentiation, was another downregulated gene found in our transcriptome. Luo et al. observed *MYC* downregulation during the differentiation process of myoblasts into myotubes [33]. Likewise, *FKBP5* was downregulated. It encodes the peptidyl-prolyl isomerase, creates the Cdk4-Hsp90 complex which blocks the cyclinD1-Cdk4 bond formation and further prevents Cdk4 phosphorylation. Therefore, *FKBP5* is responsible for myoblast differentiation and its downregulation slows down this differentiation [34].

Our transcriptome revealed downregulation of genes linked to chromosome Y, such as *DDX3Y*, *RPS4Y1*, *UTY*, *KDM5D*, which play an important role in cardiac mesoderm development [35]. *DDX3Y* encodes RNA helicase involved in the differentiation process [36], as in RNA splicing and translation processes. Furthermore, this enzyme is localized within the stress granules present in muscle cells in oxidative stress and muscle stress conditions [37,38]. *RPS4Y1* encodes a ribosomal protein, located in minor ribosomal subunit. *RPS4Y1* was found in the transcriptome profile of male muscle [39,40].

Instead, *UTY*, *UTX* paralogue, involved in the demethylation of histone H3 Lysine 27 (H3K27), in cell transcription and differentiation process [41]. *KDM5D*, encodes lysine demethylase. It plays a key role in protein synthesis and cell cycle regulation. Several studies conducted on human embryonic stem cells (hESCs) have shown that *KDM5D* is involved in cardiomyocytes differentiation [42]. Therefore, the downregulation of these genes could slow down the differentiation process.

In our transcriptomic profile, *ZFP36* was found. *ZFP36*, known as Tristetraprolin (TTP), encodes a post-transcriptional regulator. It binds the 3’UTR mRNA of many transcripts such as protein 1 for the determination of myoblasts (MyoD), preventing the translation process. Increases in MyoD levels were observed in muscles of ZFP36-deficient mice, therefore *ZFP36* hinders SCs activation. Furthermore, p38α/β MAPK pathway activation causes phosphorylation and consequent TTP inactivation. Thus, TTP inactivation enables MyoD transcript translation and promotes myoblast differentiation [43,44]. In our case, we observed an upregulation of ZFP36 which suggests that SCs may be inactivated.

Our results showed genes involved in the apoptotic process and oxidative stress. We observed *MTRNR2L8* downregulation, encoding a mitochondrial peptide, known as Humanin. A recent study believes that Humanin has a cytoprotective role in the myocardium, counteracting oxidative stress [45].

In our analysis we observed *GADD45B* downregulation, which encodes a protein involved in the apoptotic process and DNA damage repair. Experimental studies showed high Gadd45b concentrations in cardiomyocytes after ischemic injury [46]. Moreover, in catabolic diseases such as cachexia, *GADD45B* downregulation occurs and affects muscle tissue regeneration [47]. Instead, *TIPARP* also known as *PARP* was downregulated. It belongs to the poly (ADP-ribose) family of polymers, and catalyzes the transfer of the ADP-ribosyl group to different protein targets using NAD^+^. It is involved in DNA damage repair and regulates the transcriptional activity of several transcription factors such as p53 and Fork-head box transcription factor O3 (FoxO3). Interestingly, *PARP1* increase in cardiovascular diseases such as cardiac hypertrophy. Indeed, *PARP1* deficiency in mice has been shown not to induce hypertrophy. *PARP1* promotes FoxO3 dephosphorylation in the nucleus and promotes the transcription of apoptotic genes in cardiomyocytes [48,49].

*XIST* encoding a non-coding transcript (lncRNA) causes X chromosome inactivation to compensate for gene dosage between males and females. It is involved in various disease such as tumors, heart failure, and in particular cardiac hypertrophy. Other experimental studies showed *XIST* upregulation in hypertrophic rats exerts an anti-hypertrophic action in cardiomyocytes, determining a protective role [50].

Instead, we found *NR4A3* downregulation, which encodes a protein also known as neuron-derived orphan receptor (NOR-1). NOR-1 is expressed in skeletal muscle, and involved in vascular smooth muscle cell proliferation, and generally its concentration increases in response to inflammatory stimuli, causing a hypertrophic process [51,52].

At the same time, the transcriptomic analysis showed the downregulation of genes involved in muscle contraction in patients suffering from severe migraine. In particular, we found the downregulation of *TPM4* and *ACTC1* genes. *TPM4* encodes Tropomyosin 4 (Tpm4), a member of the tropomyosin family of actin-binding proteins, known to play an essential role in regulating muscle contraction [53,54]. *TPM4* defines novel filaments in skeletal muscle associated with muscle remodeling and regeneration in normal and diseased muscle [55]. The analysis of muscle biopsies confirms that Tpm4, in the longitudinal filaments of the muscles, is involved in the regeneration process of the muscle fiber. The important finding in the transcriptomic profile was the downregulation of *ACTC1*, also known as striated α-actin, and encodes a contractile protein of the sarcomere [56] located mainly in the heart but also expressed in skeletal muscle. It is downregulated in adult individuals involved in muscle contraction. Its expression changes with aging. The increase in Ca^2+^ causes tropomyosin to move from the α-actin filament, exposing its binding sites to allow interaction with myosin [57,58]. The ATP concentration increases in muscle contraction. The increase in ATP activates a signal cascade that results in an increase in Ca^2+^ and activation of Erk1/2, p38 MAPK, and mammalian target of rapamycin (mTOR) promoting *JUNB* and *IL6* transcription [59]. In our transcriptomic profile, *JUNB* was downregulated. Jun-B is present in the nucleus of mature muscle fibers, involved in maintaining muscle mass and is considered a regulator of cell proliferation. Jun-B is also involved in the protein synthesis process, especially myosin. Raffaello et al. demonstrated that Jun-B binds FoxO3 to form a complex. This binding prevents FoxO3 from interacting with atrogin-1 and Muscle RING Finger 1 (MuRF1) promoters, avoiding muscle atrophy. In particular, Jun-B and FoxO3 interaction slows FoxO3-mediated proteolysis. Thus, Jun-B deficient conditions promote protein catabolism [60]. As shown in our investigation, *TRIM63* was upregulated, encoding MuRF1, a member of the ubiquitin-ligase E3 class. In various pathological conditions, when MuRF1 is overexpressed, there is an increase in proteolysis, probably of contractile proteins, which over time degenerates into muscle atrophy [61]. Interestingly, a study by Sacheck et al. involves the administration of dexamethasone in myoblast cultures. This study highlighted MuRF-1 as an antagonist of the cell growth process and promoter of protein breakdown [62]. We observed *OTUD1* deregulation, belonging to the deubiquitinase family. This removes ubiquitin from proteins involved in inflammatory processes [63]. The role of *OTUD1* in muscle is not well known. However, its levels rise after physical activity. It is thought to be involved in the deubiquitination and stabilization of contractile proteins. *OTUD1* deficiency can destabilize the proteins involved in muscle contraction. In muscle contraction, intracellular Ca^2+^ leads to an increase in the transcription of the interleukin 6 (*IL6*) gene. *IL6* is also known as a myokine involved in the proliferation and differentiation of myoblasts [64]. STAT3 is the target of IL-6.

However, myokine deficiency causes STAT3 downregulation, and cannot activate the cyclin D1 gene [65]. Furthermore, this myokine activates STAT3 by modulating the activation of the cytokine-3 signal suppressor (SOCS3) [64]. *SOCS3* is poorly expressed in both muscle fibers and muscle stem cells, and plays a role in cell differentiation. Low *SOCS3* expression can lead to a slowing of the muscle regeneration process [66]. In relation to our results, it was possible to deduce that IL6 and SOCS3 deregulation in pathological conditions such as TMD could cause a slowing down of the muscle regeneration mechanism.

In the transcriptomic analysis of masticatory muscles, we found the *GLUL* gene downregulated. *GLUL*, known as glutamine synthetase, is an encoding enzyme that catalyzes the synthesis reaction of glutamine from glutamate and ammonia. Glutamine synthetase downregulation causes accumulation of glutamate. Castrillon et al. conducted a study on the provenance of myofascial pain in TMD patients suffering from migraines, evaluating the effects of glutamate administration in masseter and temporalis muscles. The result obtained determines an increase in myofascial pain implied by the activation of NMDA receptors [67]. Glutamate is the main component of the glutathione molecule, with antioxidant power against reactive oxygen species (ROS) [68]. *GSTM1* belongs to the glutathione-s-transferase (GST) class which plays a defensive role against ROS [69]. Tripathy et al. performed a study in migraine patients, measuring the dosage of antioxidant molecules. Their study showed low GST levels in migraine patients, which matches the result of our transcriptome. Therefore, low GST levels could implement an increase in oxidative stress [70].

In our analysis, *SLC2A3* was downregulated. It encodes glucose transporter-3 (GLUT3) [71], found at low concentrations in skeletal muscles. GLUT3 expression increases during muscle repair processes to enhance glucose uptake in muscle fibers. Several in vitro studies carried out on fetal rat myoblasts have shown a decrease in GLUT1, and an increase in GLUT3 expression during myoblasts’ fusion [72]. On the other hand, *MLXIPL*, in our transcriptome, was upregulated and encodes for Carbohydrate-responsive element-binding protein (ChREBP), a protein binding the reactive elements to carbohydrates, present to a lesser extent in skeletal muscle than in the liver. Its expression increases in response to increased glucose concentration, increased lipogenesis, and regulation of glucose homeostasis [73].

Finally, in our investigation we found a peculiar *HBB* downregulated gene, encoding B-globin, a subunit of hemoglobin. Several muscle studies have observed that decreased hemoglobin is associated with lower muscle mass, and therefore lower muscle strength [74]. Further confirmation was given by Shephard et al. who found a reduced expression of HBB in muscle mitochondria [75].

Furthermore, intense muscle activity damages blood vessels, impairing blood flow and oxygen supply, thus slowing down the muscle repair process [76]. Referring to our transcriptomic analysis of masseter and temporalis muscles in TMD patients with different degrees of migraine, it was possible to detect the downregulation of the genes involved in the mechanism of muscle regeneration.

Ultimately, our findings may suggest a slowing of muscle fiber repair processes in TMD patients with migraine.

Our transcriptomic analysis conducted on muscle biopsies obtained from TMD migraine patients indicates that the transcriptional expression of genes commonly expressed in the two experimental groups (patients with moderate versus severe migraine) does not significantly correlate with the migraine score. This represents a limitation of our study as well as the impossibility of carrying out a transcriptomic analysis on post-operative asymptomatic patients.

In conclusion, the association of innovative intervention techniques with molecular investigations could be useful for improving musculoskeletal disorders.

## Figures and Tables

**Table 1:** Anamnestic data of patients.

Variables	Patient 1	Patient 2	Patient 3	Patient 4	Patient 5
Gender	Male	Male	Female	Female	Male
Age	52	68	59	68	62
Familiar headache	No	No	No	No	No
Head Pain Perception	Bilateral	Bilateral	Bilateral	Bilateral	-
VAS	2/10	6/10	9/10	6/10	0
Migraine Attack duration (without drug)	2 h	4 h	24 h	10 h	-
Nausea	0	0	7/10	2/10	0
Vomit	0	0	6/10	2/10	0
Photophobia	0	4/10	8/10	4/10	0
Phonophobia	0	3/10	8/10	4/10	0
Osmo-phobia	2/10	0	8/10	3/10	0
Tearing	0	1/10	7/10	3/10	0
Rhinorrhea	0	0	6/10	2/10	0
Difficult moving	0	0	6/10	2/10	0
Sensory processing disorder	0	0	6/10	0	0
Dysphasia	0	0	4/10	0	0
Limitation Activity daily for headache	No	No	No	No	No
Analgesic taken in a month	1–3	1–3	4–10	4–10	0
Analgesic efficacy during migraine attack	Yes	Yes	No	Yes	-
Migraine attacks in the last three months	0	10	80	50	0
Drugs used for migraine attacks	NSAID	NSAID	NSAID	NSAID	-
Professional status	Worker	Non- worker	Non- worker	Non- worker	Worker

VAS, visual analogue scale; NSAID, non-steroidal anti-inflammatory drug. The analysis of migraine attack frequency and various symptoms in TMD patients suggests that patient 1 and patient 2 suffer from moderate migraines. Patient 3 and patient 4 suffer from severe migraines, while patient 5 has pleomorphic adenoma of the parotid and represents control.

**Table 2:** Differentially expressed genes in control or moderate against severe groups.

Gene	Name	Fold Change Control vs. Severe	Fold Change Moderate vs. Severe
*ACTC1*	actin alpha cardiac muscle 1	−2.54	−2.49
*DDX3Y*	DEAD-box helicase 3 Y-linked	−10.24	−9.82
*EGR1*	early growth response 1	N.S.	−2.23
*FKBP5*	FKBP prolyl isomerase 5	−2.98	N.S.
*FOS*	FOS proto-oncogene, AP-1 transcription factor subunit	N.S.	−3.39
*FOSB*	FOS proto-oncogene, AP-1 transcription factor subunit	−3.90	−5.44
*GADD45B*	growth arrest and DNA damage inducible beta	−3.96	−3.80
*GLUL*	glutamate-ammonia ligase	−2.20	−2.20
*GSTM1*	glutathione S-transferase mu 1	−7.80	N.S.
*HBB*	hemo-globin subunit beta	−3.73	−2.56
*IL6*	interleukin 6	N.S.	−5.96
*JUNB*	JunB proto-oncogene, AP-1 transcription factor subunit	N.S.	−3.23
*KDM5D*	lysine demethylase 5D	−10.94	−10.49
*MLXIPL*	MLX interacting protein like	2.25	N.S.
*MTRNR2L8*	MT-RNR2 like 8	−4.26	−6.25
*MYC*	MYC proto-oncogene, bHLH transcription factor	N.S.	−2.50
*NR4A3*	nuclear receptor subfamily 4 group A member 3	−3.78	−5.85
*OTUD1*	OTU deubiquitinase 1	−3.26	−3.51
*RPS4Y1*	ribosomal protein S4 Y-linked 1	−12.23	−12.05
*SLC2A3*	solute carrier family 2 member 3	−4.36	−4.23
*SOCS3*	suppressor of cytokine signalling 3	N.S.	−4.50
*TIPARP*	TCDD inducible poly(ADP-ribose) polymerase	−3.05	−2.89
*TPM4*	tropomyosin 4	−3.19	−3.61
*TRIM63*	tripartite motif containing 63	N.S.	−2.37
*UTY*	ubiquitously transcribed tetratricopeptide repeat containing, Y-linked	−11.61	−11.08
*XIST*	X inactive specific transcript	9.41	9.37
*ZFP36*	ZFP36 ring finger protein	N.S.	−2.92

Statistically significant differentially expressed genes with q-value lower 0.05 and absolute fold change higher than 2. “N.S.” is used if a gene does not differ in a statistically significant manner.

## Data Availability

The data presented in this study are openly available in the NCBI Sequence Read Archive with the reference number PRJNA699558.
